# RESILIENCE AND MULTIFACTORIAL STRESSORS AMONG OLDER ADULTS DURING THE COVID-19 PANDEMIC: A QUALITATIVE STUDY

**DOI:** 10.1093/geroni/igac059.366

**Published:** 2022-12-20

**Authors:** Melissa Hladek, Deborah Wilson, Sabrina Shofner, Alden Gross, Karen Bandeen-Roche, Nancy Schoenborn

**Affiliations:** Johns Hopkins University, Baltimore, Maryland, United States; Johns Hopkins University School of Nursing, Baltimore, Maryland, United States; Johns Hopkins University, Baltimore, Maryland, United States; Johns Hopkins Bloomberg School of Public Health, Baltimore, Maryland, United States; Johns Hopkins Bloomberg School of Public Health, Baltimore, Maryland, United States; Johns Hopkins University, Baltimore, Maryland, United States

## Abstract

The COVID-19 pandemic represents a complex stressor that is experienced differently across individuals and age strata. The present study explored perceptions and experiences of older adults within the domains of health, social interactions, finances and care of existing chronic medical conditions; and strategies used to cope with these stressors. We recruited 30 people (mean age 81.4 years) stratified by frailty status to complete semi-structured interviews about what changes to the above domains had occurred and what coping strategies were utilized. Using inductive and deductive coding techniques, thematic analysis revealed three overarching themes. The first was Pandemic Experience, which was perceived as stressful, especially in the domains of social isolation from friends and family and concern for others’ well-being. The second domain was Resilience where participants reported highly adaptable and creative ways to connect with others and viewed the pandemic from a lens of lifetime experience, which acted as a stress buffer. The third theme was Silver Linings where participants reported unexpected renewal like reconnecting with family and friends in more meaningful ways and reconnecting with nature. We found no meaningful distinction in experience by frailty status and explore reasons for this. Policy implications including internet access and training and societal aging biases are discussed in the context of aging and coping theories.

